# A Request from the State Health Department

**Published:** 1918-08

**Authors:** William A. Davis

**Affiliations:** State Registrar of Vital Statistics


### ITEMS OF GENERAL INTEREST.

### A Request From the State Health Department.

Mrs. F. E. Daniel,
Editor *Texas Medical Journal,*
Austin, Texas.

Dear Madam: I have today issued four certified copies of birth certificates to be used in the settlement of claims for the allowance due to the children of soldiers. Numerous requests for copies are made where certificates have not been recorded.

The Federal Government accepts, and to a certain extent demands, a certified copy in the settlement of such claims. Where the birth has not been registered, the Charitable Committees and Organizations have to care for the child until its allowance is received. Such organizations are now overworked, because of war conditions.

PLEASE CALL ON ALL ETHICAL PHYSICIANS TO GO OVER THEIR BOOKS AND FILE CERTIFICATES FOR BIRTHS NOT ALREADY REGISTERED. THEY OWE THIS SERVICE NOT ONLY TO THE INNOCENT CHILD, BUT TO SUCH COMMITTEES AND ORGANIZATIONS AS WELL AS THE COMMUNITIES WHICH MUST FURNISH FINANCIAL AID TO THESE UNFORTUNATES.

The State Bureau will accept any certificate regardless of date. If the birth occurred prior to the passage of the present law, an affidavit of the physician must be attached. Printed affidavits for this purpose will be furnished free to physicians.

Yours fraternally,

WILLIAM A. DAVIS, M. D.,
*State Registrar of Vital Statistics.*

